
JOURNAL INFORMATION
==============================
NLM Title Abbreviation: ScientificWorldJournal
Journal ID: ScientificWorldJournal
NLM Journal ID: 101131163
Journal ID: TSWJ

The Scientific World Journal

ISSN: 2356-6140
EISSN: 1537-744X
Publisher: Wiley

ARTICLE INFORMATION
==============================
PMCID: PMC6084725
PMID: 30147507
DOI: 10.1100/tsw.2001.178
Article ID: 825706
Article version: 1
Subjects: Short Report

Granzyme B Induces Bid-Mediated Cytochrome C Release and Mitochondrial Permeability Transition

Alimonti Judie B. *
Shi Lianfa F.
Baijal Priti K.
Greenberg Arnold H.
Manitoba Inst. of Cell Biology University of Manitoba, Winnipeg, Man. R3E 0V9, Canada
*Judie B. Alimonti: judieali@yahoo.com
Publication date: 2001
Electronic publication date: 2001 Oct 27
Volume: 1
First page: 49
Last page: 49
Copyright: Copyright © 2001 Judie B. Alimonti et al.
Copyright year: 2001
License: This is an open access article distributed under the Creative Commons Attribution License, which permits unrestricted use, distribution, and reproduction in any medium, provided the original work is properly cited.
License URL: https://creativecommons.org/licenses/by/3.0/

Funding: National Cancer Institute Medical Research Council of Canada Manitoba Health and Research Council

==============================
Miami Nature Biotechnology Short Reports
TheScientificWorld (2001) 1(S3), 49SR
ISSN 1532-2246; DOI 10.1100/tsw.2001.178

### GRANZYME B INDUCES BID-MEDIATED CYTOCHROME C RELEASE AND MITOCHONDRIAL PERMEABILITY TRANSITION

Judie B. Alimonti*, Lianfa F. Shi, Priti K. Baijal, and Arnold H. Greenberg

675 McDermot Ave., Manitoba Institute of Cell Biology, Winnipeg, MB, Canada, R3E 0V9
* judieali@yahoo.com

**INTRODUCTION.** Granzyme B (GrB) is an aspartyl serine protease located in the granules of cytotoxic T cells (CTL) and natural killer (NK) cells that induce apoptosis. Recent work suggests that mitochondria play a key role in the regulation of cell death in mammalian cells through the release of cytochrome c (CC), as well as the loss of the mitochondrial membrane potential ($\Delta\Psi m$)[1]. GrB has been shown to induce mitochondria to release CC that interacts with Apaf-1 and caspase 9 resulting in the activation of effector caspases and the subsequent apoptotic biochemical and morphological cellular changes (2,3). However, the exact mechanism of cytochrome *c* release remains controversial and unresolved.

We will demonstrate that GrB mediates CC release from isolated mitochondria in vitro through an S100 protein that we identify as the BCL-2 family member BID. Furthermore, GrB in the absence of BID or other S100 proteins can open the mitochondrial PT pore with the loss of $\Delta\Psi m$. Thus, we have identified two mechanisms by which GrB disrupts mitochondrial function and promotes cell death.

**METHODS.** We utilized a cell free *in vitro* assay where isolated mouse liver mitochondria were treated with GrB in the presence or absence of the cytosolic S100 fraction. We examined GrB effects on the mitochondria by i) using western blotting for cytochrome c release, and ii) by fluorescent Calcein AM and TMRM staining to examine the PT and loss of $\Delta\Psi m$, respectively.

**RESULTS.** We determined in a cell free assay that GrB-mediated CC release requires a protein in the cytosolic S100 fraction (Fig.1A). The protein was purified from the S100 fraction on sequential columns (Hydroxyapetite, Phenylsuperose, then Mono Q). We determined that the CC releasing fractions from the mono Q column corresponded exactly to the fractions containing the pro-apoptotic molecule BID (Fig.1B). S100 depleted of BID failed to cause GrB-induced CC release in isolated mitochondria. Also, when recombinant BID was added to mitochondria there was CC release in the absence of S100 suggesting only BID from the cytosol is required for CC release.

Fig. 1

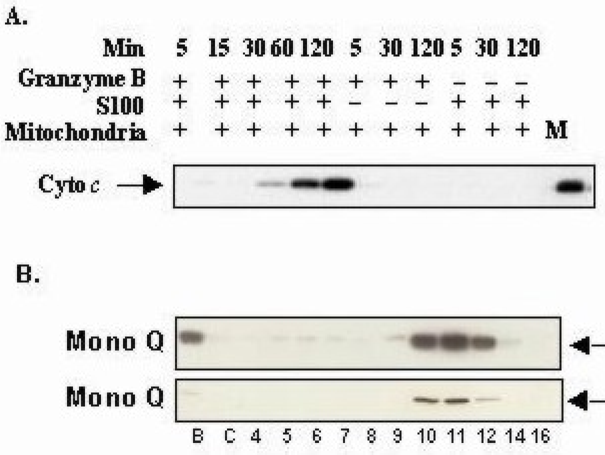

Fig. 2

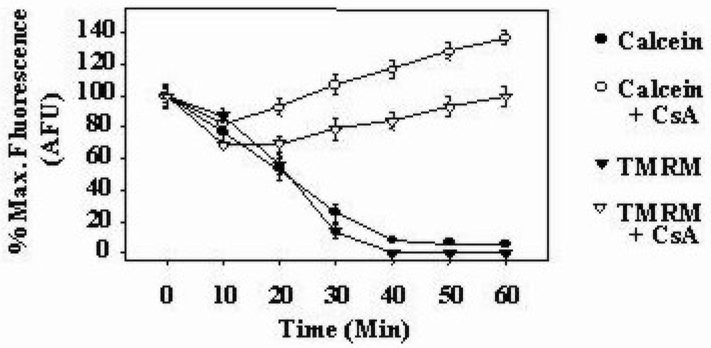

GrB also mediated ΔΨm suppression in isolated mitochondria using the TMRM potentiometric fluorescent dye, and caused the decrease in ΔΨm in the absence of the S100 fraction suggesting GrB acts on a mitochondrial protein directly. Pre-incubating mitochondria with the permeability transition pore inhibitor cyclosporin A (CsA) blocked both permeability transition (calcein AM fluorescent staining) and ΔΨm loss (TMRM) indicating a link between PT and ΔΨm (Fig.2).

**DISCUSSION.** We investigated how GrB effects mitochondria utilizing an *in vitro* cell free system and determined that CC release and PT are initiated by distinct mechanisms. The cleavage of cytosolic BID by GrB results in truncated BID initiating mitochondrial CC release. BID is the sole cytosolic protein responsible for this phenomenon in vitro, yet caspases were found to participate in CC release in some cells. On the other hand, GrB acts directly on mitochondria in the absence of cytosolic S100 proteins to open the permeability transition pore and disrupt the ΔΨm. We suggest that GrB acts by two distinct mechanisms on mitochondria that ultimately lead to mitochondrial dysfunction and cellular demise.

**ACKNOWLEDGEMENT.** We thank Drs. J. Yuan, A. Degertev, T. Ley, R. Hakem, T. Mak for their gifts of reagents. This work is supported by the National Cancer Institute of Canada, the Medical Research Council of Canada, Manitoba Health and Research Council, and the George H. Sellers Foundation.

**REFERENCES.**

1. MacDonald, G., Shi, L.F., Velde, S.V., Lieberman, J., and Greenberg, A.H. (1999) J. Exp. Med. 189, 131-143
2. Rodriquez, J. and Lazebnik, Y. (1999) Genes Dev. 13, 3179-3184
3. Li, P., Nijhawan, D., Budihardjo, I., Srinivasula, S.M., Ahmad, M., Alnemri, E.S., and Wang, X.D. (1997) Cell 91, 479-489
